# The Botrytis cinerea xylanase Xyn11A contributes to virulence with its necrotizing activity, not with its catalytic activity

**DOI:** 10.1186/1471-2229-10-38

**Published:** 2010-02-25

**Authors:** Judith Noda, Nélida Brito, Celedonio González

**Affiliations:** 1 Departamento de Bioquímica y Biología Molecular, Universidad de La Laguna, E-38206 La Laguna (Tenerife), Spain

## Abstract

**Background:**

The *Botrytis cinerea *xylanase Xyn11A has been previously shown to be required for full virulence of this organism despite its poor contribution to the secreted xylanase activity and the low xylan content of *B. cinerea *hosts. Intriguingly, xylanases from other fungi have been shown to have the property, independent of the xylan degrading activity, to induce necrosis when applied to plant tissues, so we decided to test the hypothesis that secreted Xyn11A contributes to virulence by promoting the necrosis of the plant tissue surrounding the infection, therefore facilitating the growth of this necrotroph.

**Results:**

We show here that Xyn11A has necrotizing activity on plants and that this capacity is conserved in site-directed mutants of the protein lacking the catalytic activity. Besides, Xyn11A contributes to the infection process with the necrotizing and not with the xylan hydrolyzing activity, as the catalytically-impaired Xyn11A variants were able to complement the lower virulence of the *xyn11A *mutant. The necrotizing activity was mapped to a 30-amino acids peptide in the protein surface, and this region was also shown to mediate binding to tobacco spheroplasts by itself.

**Conclusions:**

The main contribution of the xylanase Xyn11A to the infection process of *B. cinerea *is to induce necrosis of the infected plant tissue. A conserved 30-amino acids region on the enzyme surface, away from the xylanase active site, is responsible for this effect and mediates binding to plant cells.

## Background

*Botrytis cinerea *is a phytopathogenic fungus with a wide host range and a necrotrophic life style (for a review see [1-3]). As part of its invasion strategy, *B. cinerea *and other necrotrophs are thought to promote programmed cell death (PCD), or apoptosis, in plant cells surrounding the lesion by making use of the plant defence response known as the hypersensitive response (HR) [4]. HR comprises a range of effects triggered by pathogens that culminate in PCD of the plant cells around the infected area [5]. It is an effective defence against biotrophs, preventing the progression of the infection, but it has been suggested that HR can be exploited by necrotrophs, such as *B. cinerea*, for its own benefit [1-4]. The basic idea is that necrotrophs produce signals able to induce plant cells to kill themselves and then grow on the dead tissue.

Several *B. cinerea *derived metabolites and proteins have been shown to cause cellular death when applied to plant cells or tissues, like the small compounds botrydial and botcinolide [6], Oxalic acid [2,7], enzymes with endopolygalacturonase activity [8] and Nep1-like proteins (NLPs) [9]. Only in the case of oxalic acid and NLPs, the mechanisms of toxicity were studied, and evidences were presented in both instances supporting the induction of programmed cell death. In the case of cell wall degrading enzymes causing plant cell death, such as endopolygalacturonases [8], the doubt always arises if the actual inducers of cell death are the enzymes themselves, or the products of their activity. The latter seems to be the case, for example, for the *B. cinerea *endopolygalacturonase 2, since point mutations in the protein that abolish its enzymatic activity also eliminate its necrosis inducing ability [8].

We have previously shown that the secreted endo-β-1,4-xylanase Xyn11A is required for full virulence in *B. cinerea*, since the mutation of the corresponding gene by gene replacement greatly reduced virulence in tomato leaves and grape berries [10]. Moreover, reintroduction of the wild-type *xyn11A *gene into the *xyn11A *knock-out mutants completely restored the wild-type phenotype. These results were difficult to explain on the sole basis of the modest reduction in xylanase activity observed for the mutants, 30%, especially if one takes into account that the plant tissues for which a reduction in virulence was observed are poor in xylan. An alternative hypothesis we proposed at that time was the possibility that Xyn11A was contributing to virulence not with its xylanase activity, but with a putative necrosis inducing activity that had been observed for two xylanases from other fungi, *Trichoderma reesei *xylanase II [11] and *Trichoderma viride *EIX [12]. This way, Xyn11A would act by killing the plant tissue surrounding the infected area and therefore would allow *B. cinerea to *grow faster on dead tissue. Here we verify this hypothesis and show that the contribution of Xyn11A to virulence does not rely on its enzymatic activity, but rather on its ability to elicit necrosis in plants.

## Results

### Expression and purification of Xyn11A in *Pichia pastoris*

**Figure 1:**
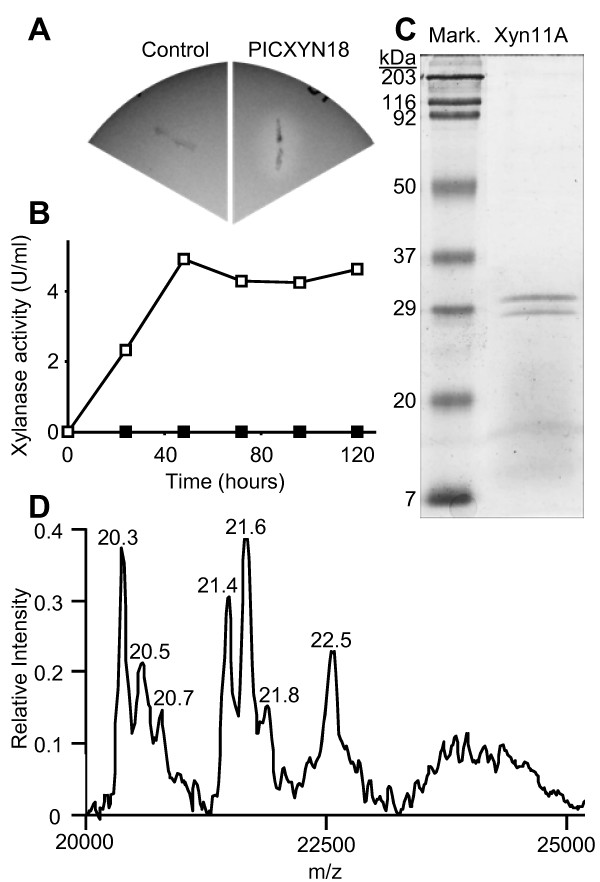
**Expression of the xylanase Xyn11A in *Pichia pastoris***. PICXYN18 is the yeast strain transformed with the *xyn11A *cDNA in plasmid pPIC3.5. Control was transformed with pPIC3.5 vector alone. **A**) Xylan degradation halo produced on plates by the strain expressing Xyn11A. **B**) Xylanase activity in the culture supernatant of PICXYN18 (white square) or control (black square) strains, induced with methanol. **C**) SDS-PAGE of purified Xyn11A. **D**) SELDI-TOF spectrum for the purified Xyn11A showing the molecular weight (kDa) of the protein isoform in each peak.

The yeast *Pichia pastoris *was transformed with the *xyn11A *cDNA under the control of the *AOX1 *promoter to induce the production of Xyn11A by methanol and its secretion by making use of its own signal peptide. Yeast transformant PICXYN18 showed abundant xylanase secretion in plates and in liquid culture and was selected for all subsequent experiments (Fig. 1A and 1B). Supernatant from a methanol-induced culture of this transformant showed two new polypeptides having masses around that predicted for the mature Xyn11A [10], 20.6 kDa. These expression products were then purified from the culture medium by a two-step protocol consisting of differential ammonium sulphate precipitation and gel exclusion chromatography. Three chromatographic fractions showed high xylanase activity and the presence of just the two new polypeptides by SDS-PAGE (Fig. 1C). These three fractions were pooled and dialyzed against water overnight and the resulting purified Xyn11A showed a protein concentration of 46 μg/ml and a specific activity of 122.7 U/mg protein. In order to check if the two protein bands observed in the purified xylanase fraction were both the product of the *xyn11A *gene, the two protein bands were cut from the gel and were subjected to peptide mass fingerprinting at the proteomic facility of the Centro Nacional de Biotecnología http://proteo.cnb.csic.es. Both bands were identified as the same protein, Xyn11A. Moreover, we used SELDI-TOF mass spectrometry to analyze the purified xylanase fraction and, surprisingly, the heterogeneity of the purified xylanase was higher than expected (Fig. 1D), with at least 7 different species differing slightly in mass. The reason for this phenomenon may be differences introduced by the *Pichia *glycosylation system from molecule to molecule [13,14], or alternatively an incomplete processing by *Pichia *of the putative propeptide in the protein, as has been observed before for other proteins [15,16].

The purified enzyme was characterized and the kinetic parameters were determined. We estimated both the optimal temperature, by carrying out the enzymatic reactions at different temperatures, and the thermal stability of Xyn11A, by assaying residual activity after incubation at different temperatures for 1, 2, 4, 10 or 15 minutes. The optimal temperature was about 45°C, but the enzyme lost activity rapidly above 35°C (not shown). Since the activity measured at 40 or 45°C seems to be the product of a decreasing quantity of a very active enzyme, the assay temperature chosen for all future incubations was 35°C. Concerning the effect of the pH, Xyn11A showed an optimal activity at approximately pH 5.0, in contrast to the extremely high predicted pI for the mature enzyme of 9.1 [10], but in accordance with the usually moderately acid pH of the *B. cinerea *extracellular medium [17], and the enzyme was very stable from pH 3 to 7 up to 4 hours , at room temperature (not shown). By using the optimized assay conditions, we calculated the Km of the enzyme for the substrate beechwood xylan resulting in a value, 7.1 g/l, in the same range as what has been found for other fungal xylanases [18].

### Xyn11A has necrotizing activity on tomato and tobacco leaves

**Figure 2:**
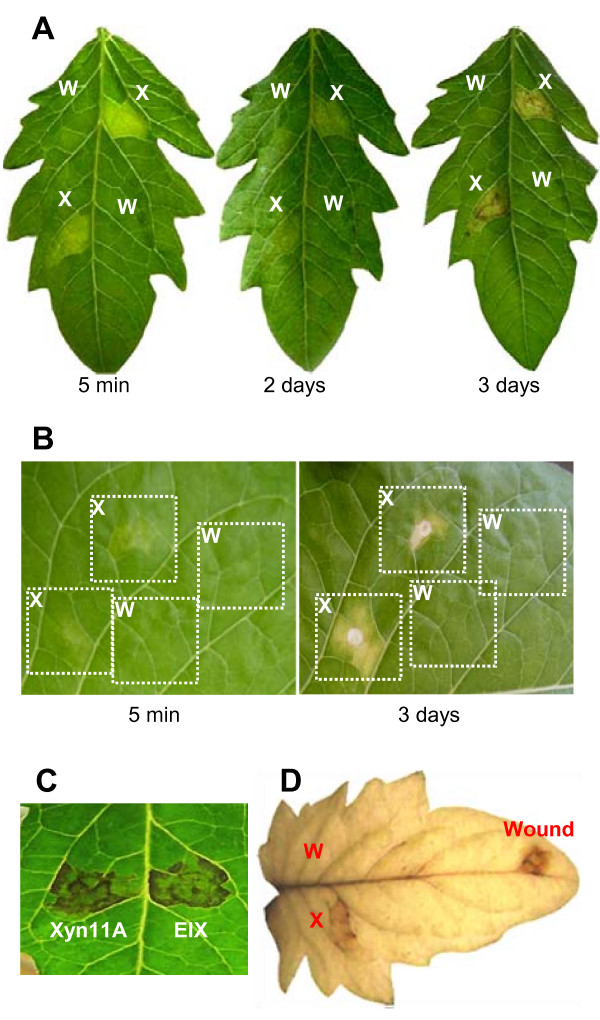
**Necrotizing and H_2_O_2_-inducing activities of Xyn11A**. **A**) Infiltration in tomato (cv. Moneymaker) leaves. **B**) Infiltration in tobacco (cv. Havana) leaves. **C**) Side-by-side comparison of necrotizing activity of Xyn11A and EIX on tomato (cv. Moneymaker). Picture was taken 4 days after infiltration. **D**) Infiltration of tomato leaves and treatment with DAB to reveal H_2_O_2_. A positive control was made by wounding the leaf. X: xylanase Xyn11A; W: water; EIX: xylanase EIX from *Trichoderma viride*.

In order to check if, as previously hypothesised, Xyn11A was able to induce necrosis on plants, the purified enzyme dissolved in water at a concentration of 70 μg/ml was infiltrated in tomato and tobacco leaves and its effect was recorded for several days (Fig. 2A and 2B). The area of the leaf treated with Xyn11A became necrotic about 2-5 days after infiltration, but this effect was absent in areas infiltrated with water or with the control protein Bovine Serum albumin (not shown). Positive controls made using the commercially available xylanase EIX from *T. viride *[12] caused similar effects as those caused by Xyn11A (Fig. 2C). Both the appearance of the lesions as well as its time course were similar to what has been previously reported for the xylanases from *T. viride *and *T. reesei *[11,19,20].

The ability of Xyn11A to induce the production of reactive oxygen species (ROS) in the infiltrated leaves was also studied by determining the production of hydrogen peroxide with diaminobenzidine (DAB), since H_2_O_2 _is one of the landmarks of the hypersensitive response [5]. Leaves were first infiltrated with purified Xyn11A as before, then treated with DAB as explained in materials and methods, and finally, decolorized with ethanol to allow easier visualization of the dark, reduced DAB precipitate (Fig. 2D). A clear brown precipitate was observed only in the leaf areas that had been infiltrated with Xyn11A but not in those not infiltrated or infiltrated with water. Positive controls were made by infiltration with *T. viride *EIX xylanase (not shown) and by wounding the tip of leaves, which has been shown previously to induce the production of ROS [21], and the response obtained in both cases was similar to that obtained for Xyn11A.

**Figure 3:**
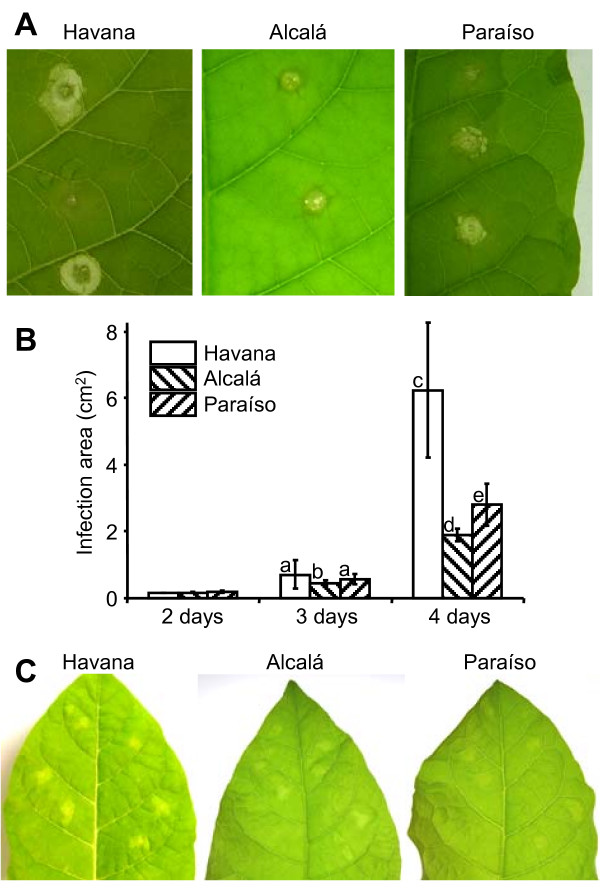
**Differences in susceptibility to *B. cinerea *and to the isolated Xyn11A protein shown by three *N. tabacum *cultivars: cv. Havana, cv. Alcalá, and cv. Paraíso**. **A**) Example of the infection caused by *B. cinerea *in the three cultivars. The fungal strain used was B05.10 (wild-type). Pictures were taken 3 days after inoculation. **B**) Mean infection areas obtained at different times after inoculation, calculated from at least 15 infections. **C**) Necrosis inducing activity of purified Xyn11A on the three cultivars. Pictures were taken 4 days after infiltration. Bars marked with different letters are statistically different (P < 0.05 by Student's t test).

The ability of Xyn11A to induce necrosis was cultivar dependent in the case of tobacco. Three tobacco cultivars differing in their susceptibility to *B. cinerea *were assayed for their capacity to develop necrosis after infiltration with Xyn11A, *Nicotiana tabacum *cv. Havana, and two local varieties, Alcalá and Paraíso. The diameter of infection areas for the two local varieties were about half the value obtained for Havana, and similarly, Xyn11A induced a response when infiltrated in the leaves of the Havana cultivar that was much stronger than that obtained for the other two (Fig. 3).

### Necrotizing activity of Xyn11A is independent of its xylanase activity

**Figure 4:**
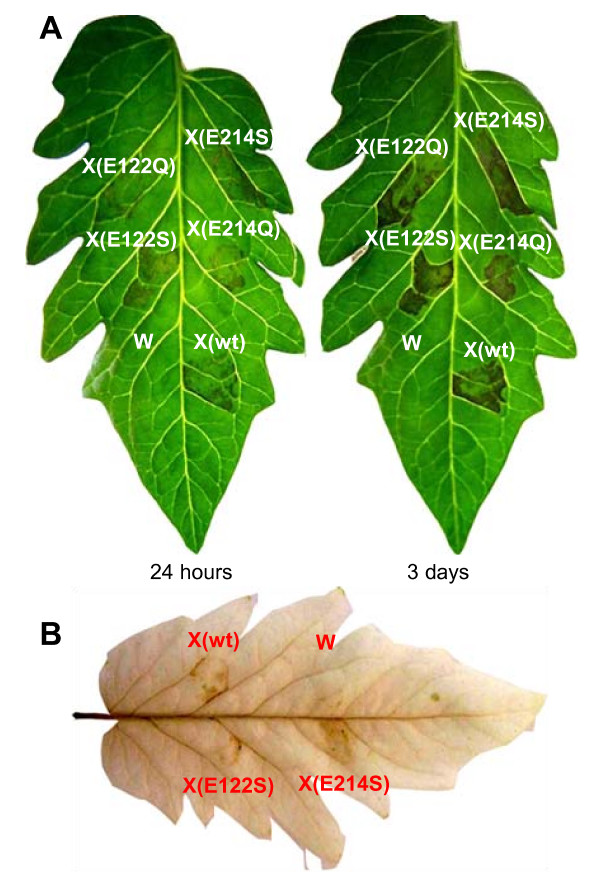
**Effect of mutations affecting the Xyn11A xylan-hydrolysis active site on its necrotizing and H_2_O_2_-inducing activities on tomato (cv. Moneymaker) leaves**. Development of necrotic lesions (**A**) and production of H_2_O_2 _(**B**) in the leaves by infiltration with wild-type and site-directed mutant Xyn11A. X: infiltration with wild-type xylanase (wt) or the indicated non-xylan-hydrolyzing mutant proteins. W: Control infiltration with water.

One obvious question at this point was if the necrosis inducing activity of Xyn11A and its enzymatic activity on xylan were independent properties of the enzyme, what would rule out the possibility that the actual inducers of necrosis were xylan oligomers. This has already been proven for the necrosis inducing xylanases from *T. reesei *[11] and *T. viride *[22], and in the latter case the eliciting epitope has been mapped to a region of the enzyme surface that is away from the catalytic site [23]. In order to check if this is also true for Xyn11A, we generated four different mutants of Xyn11A in which either one of the two glutamic acid residues in the active site that are essential for the xylanase activity was substituted by either Gln or Ser. The four mutant proteins were expressed in *P. pastoris *and purified as explained before for the wild-type Xyn11A. All four proteins were unable to degrade xylan (not shown), but retained the same necrotizing activity as the wild-type, as well as the ability to induce the production of H_2_O_2 _(Fig. 4). These results confirm that in order to induce the development of necrotic lesions in plant tissues, Xyn11A does not need to be able to hydrolyze xylan.

### The xylanase activity of Xyn11A is not necessary to complement the *xyn11A *mutant phenotype in *B. cinerea*

**Figure 5:**
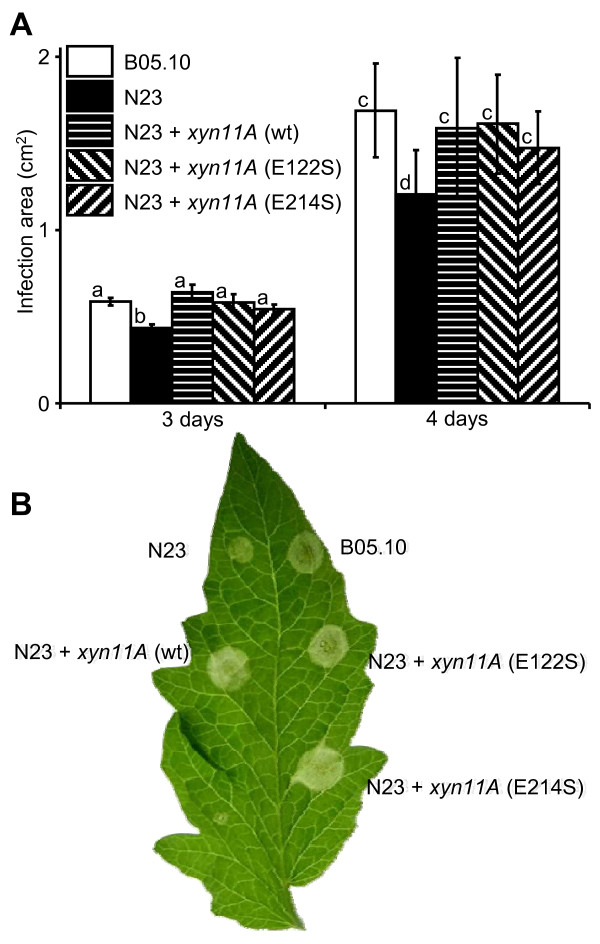
**Complementation of the *xyn11A *mutation in *B. cinerea *with the wild-type *xyn11A *gene and the site-directed mutant alleles coding for non xylan-hydrolyzing proteins**. **A**) Mean infection areas on tomato leaves generated by the wild-type strain B05.10, the *xyn11A *mutant N23, and N23 retransformed with either the wild-type *xyn11A *gene (wt) or the indicated site-directed mutated genes. Mean areas were calculated from at least 50 infections. **B**) Example leaf of the experiment in (**A**), 2 days after inoculation. Bars marked with different letters are statistically different (P < 0.05 by Student's t test).

The lack of the protein Xyn11A in *B. cinerea *makes the fungus less virulent than the wild-type [10]. Due to the fact that the two *xyn11A *mutants already available [10] showed a somehow variable phenotype with respect to virulence, we generated 6 new *xyn11A *mutants by transforming the wild-type strain B05.10 with the same construction used before [10]. All of the new mutants were characterized by Southern-blot and PCR as having a single integration of the foreign DNA at the *xyn11A *locus, similarly to the previous ones [10]. The virulence was assayed for these 6 new mutants and it was shown again that the deletion of *xyn11A *resulted in a decrease in virulence. Fig. 5 shows the reduction of infectivity for one of these new mutants, N23, which was used for the rest of the work. As discussed above, one of the hypothesis that could explain this effect is a contribution of Xyn11A to induce death of the plant cells surrounding the infected area. If this is true, and taking into account that the necrotizing and the xylanase activities are independent, then the point-mutated *xyn11A *genes coding for proteins with no xylanase activity should be able to complement the *xyn11A *mutation in *B. cinerea*, reverting the phenotype back to full virulence. In order to check if this is the case, three plasmids were generated (pNRXYN, pNRX122S and pNRX214S), all containing the nourseothricin resistance cassette along with the whole *xyn11A gene*, including the 5' and 3' untranslated regions, in three variants: the wild-type gene or an altered gene coding for one of the two site-directed mutant proteins described above, E122S and E214S. The three plasmids were transformed into the *xyn11A *mutant N23 and hygromycin and nourseothricin-resistant transformants were purified by single conidia isolation and checked by PCR for the presence of the transforming *xyn11A *gene. The oligonucleotides used were TX-Sal (5'-ACCAAGCAAGATACCAAAGTC-3') and MUT-X-XY (5'-AATCCGCGAGTCTGGATC-3') and amplified a 2.3-kb region containing the whole *xyn11A *ORF plus 1 kb and 0.5 kb of the 5' and 3' untranslated regions, respectively. This fragment can arise only from the foreign transforming DNA since the original *xyn11A *copy had been interrupted by a 2.7-kb hygromycin resistance cassette. A second PCR was made to corroborate the persistence of this interrupted *xyn11A *copy already present in the N23 mutant. This time the oligonucleotides used were MUT-X-H (5'-TCGATGCGACGCAATC-3') and MUT-X-XY (5'-AATCCGCGAGTCTGGATC-3'), which bind, respectively, to the hygromycin resistance cassette and to the *xyn11A *gene. This PCR would generate a 1.7-kb fragment only if the original *xyn11A *locus is still interrupted with the hygromycin resistance cassette. It was done to rule out a double recombination at the *xyn11A *locus that may generate a wild-type *xyn11A *gene from the copy interrupted by the hygromycin cassette and the transforming copy with the site-directed mutation. 3 to 4 transformants were identified for the three transformations that fulfilled these requirements and all of them were assayed for their virulence on tomato leaves. Representative results are shown in Fig. 5. Although with differences among individual transformants, all of them were more virulent than the *xyn11A *mutant N23 and close to the wild-type strain B05.10. These results clearly indicate that Xyn11A is contributing to virulence with its necrotizing activity and not with its xylanase activity, since the two mutant proteins had been previously shown to be unable to degrade xylan, but to retain the necrotizing activity when expressed in *P. pastoris *(Fig. 4).

**Figure 6:**
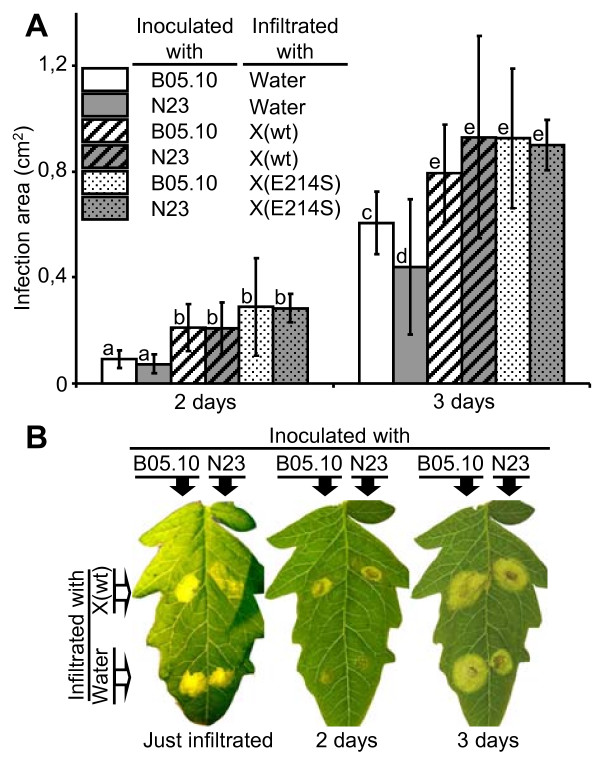
**Complementation of the *xyn11A *mutation in *B. cinerea *by the exogenous addition (by infiltration) of wild-type or non-xylan-hydrolyzing mutant Xyn11A proteins**. The indicated proteins were infiltrated in tomato leaves 4 hours before inoculation with conidia of the indicated strains. **A**) Infection areas generated by the wild-type strain B05.10 and the *xyn11A *mutant N23, inoculated on spots previously infiltrated with wild-type (wt) or mutant (E214S) Xyn11A (X) proteins. Mean areas were calculated from at least 5 infections. **B**) Example leaf of the experiment in (**A**). Bars marked with different letters are statistically different (P < 0.05 by Student's t test).

The contribution to virulence of the non-xylan-degrading Xyn11A proteins was also assayed in a different way, by exogenously providing the pure proteins to the infection process. Firstly, the wild-type Xyn11A protein and the mutant protein E214S were infiltrated in tomato leaves. Four hours later, the leaves were cut and the infiltrated areas were infected with the wild-type *B. cinerea *strain B05.10 or the *xyn11A *mutant strain N23. The presence of the Xyn11A protein, with or without xylanase activity, enhanced considerably the progression of the infection for both the wild-type and the *xyn11A *mutant strains (Fig. 6). The exogenous presence of Xyn11A in the leaves complements the lack of the protein in the mutant N23, since the differences between the wild-type and the mutant disappear. Again, this enhancing effect of Xyn11A is independent of the xylanase activity as the same effect could be seen with the Xyn11A protein devoid of xylan hydrolyzing ability.

### A 30-amino acids peptide in the Xyn11A surface mediates necrotizing activity and binding to plant cell membrane

**Figure 7:**
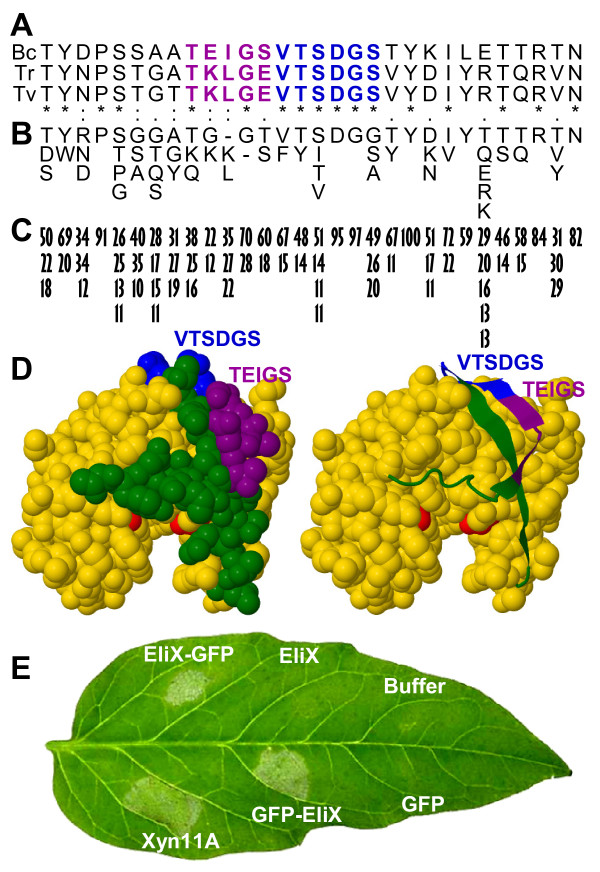
**Identification of a 30 amino acids necrosis inducing peptide in the Xyn11A surface**. **A**) Alignment of a short region (residues 131 to 160 in the immature protein) on the enzyme surface for *B. cinerea *Xyn11A (Bc) and the two other xylanases with necrosis inducing activity: *T. reesei *xylanase II (Tr) and *T. viride *EIX (Tv). Previously reported necrosis-inducing region (purple) and a well conserved contiguous region (blue) are indicated. The whole shown region was expressed in *E. coli*. **B**) Amino acids having a frequency of more than 10%, at their respective positions, in the alignment of 308 family-11 glycosyl hydrolases downloaded from Pfam. **C**) Percentage frequency on the alignment of the residues indicated in (**B**). **D**) Predicted 3D structure for Xyn11A showing (green, purple, and blue) the two beta sheets expressed in *E. coli *and the two Glu residues in the active site (red). **E**) Necrosis inducing activity of the 30-amino acids peptide expressed in *E. coli *by itself (EliX), fused to the C terminus of GFP (GFP-EliX), or fused to its N terminus (EliX-GFP). Controls were made with the whole Xyn11A, GFP alone, or buffer. Picture was taken 3 days after infiltration.

The necrotizing activity of the EIX xylanase from *T. viride *was previously mapped to the peptide TKLGE in the enzyme's surface [23]. However, this peptide is not present in Xyn11A, and is substituted by the peptide TEIGS (residues 139 to 143 in the immature protein) (Fig. 7A). The evidences presented by Rotblat et al. [23] to sustain the role of TKLGE were mainly two: first, affinity purified antibodies against the peptide blocked EIX necrotizing activity and its binding to plant cells, and second, mutant EIX in which TKLGE had been substituted by VKGT lost the necrotizing activity, but not the xylanase activity. From our point of view, it may also be possible therefore that the antibody binding, or the mutation of TKLGE, blocks the function of a bigger necrotizing epitope of which TKLGE is a part. In this respect, it is interesting that TEIGS in Xyn11A is followed by a region of 6 amino acids, VTSDGS, that is very well conserved in family 11 of glycosyl hydrolases and is located also on the enzyme surface (Fig. 7C and 7D). VTSDGS is perfectly conserved in the 3 xylanases that have been shown to induce necrosis (Fig. 7A), those of *T. viride*, *T. reesei*, and *B. cinerea*. The analysis of the alignment of 308 members of the Pfam family "Glycosyl hydrolases family 11", which are all putative xylanases, revealed that these 6 amino acids are also well conserved across the family. The first five are present in more than half of the proteins and the dipeptide Asp-Gly is present in virtually all members (Fig. 7B and 7C). The recognition by plants of a very well conserved epitope in family-11 xylanases would agree with the idea that pathogen associated molecular patterns recognized by the plant immune system should be, in principle, conserved microbial features [24]. We expressed in *Escherichia coli *a 30-aa region comprising two consecutive beta-sheets on the enzyme surface, one of which displays the region TEIGSVTSDGS (Fig. 7D). This peptide was expressed both as a fusion to the green fluorescent protein (GFP) (either at the N-terminus or at the C-terminus) and by itself, by using the pRSET series of expression vectors (Invitrogen, http://www.invitrogen.com). The three proteins were then purified with Nickel columns and infiltrated in tomato leaves to assay their elicitation ability (Fig. 7E). The two GFP fusion proteins were able to induce necrosis when infiltrated on leaves, while infiltration with GFP alone (Roche, http://www.roche-applied-science.com) dissolved in the same buffer, or with the buffer alone, did not show any effect. These results clearly indicate that the 30-aa epitope is sufficient to induce a response in the plant leading to the cell death. However, the epitope by itself did not cause any response (Fig. 7E). This difference in the activities of the peptide and its fusion with GFP may be attributed to a reduced stability of the isolated peptide or may indicate that the eliciting molecule needs to have a minimum size in order to produce any effect.

**Figure 8:**
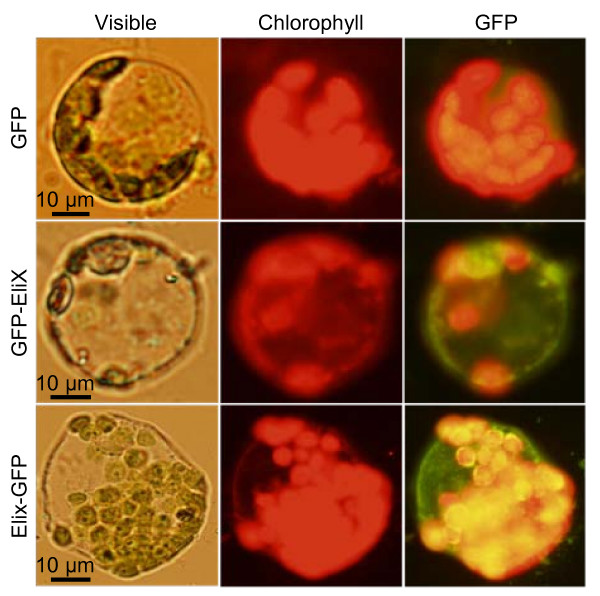
**Binding of the 30-amino acids necrosis inducing region to the plant cell surface**. Tobacco protoplast were incubated with the elicitor region fused to either end of GFP (GFP-EliX, EliX-GFP), or with GFP alone as a control, and then observed with a fluorescence microscope under visible light or under the appropriate UV light to reveal either chlorophyll or GFP.

We used tobacco spheroplasts to check the binding of the necrotizing epitope-GFP fusion proteins to the cellular membrane. Spheroplasts from *Nicotiana tabacum *cv. Havana were mixed with the two fusion proteins (or GFP alone as a negative control), incubated for 30 min at room temperature, and finally examined by fluorescence microscopy (Fig. 8). We could observe for both epitope-GFP fusions the appearance of green fluorescence in the cells, which could not be observed for the spheroplasts treated with GFP alone or for the untreated ones, indicating that the 30-aa region is sufficient for binding to the plant surface.

## Discussion

In this work we have expressed the xylanase Xyn11A from *B. cinerea *in *Pichia pastoris *and we have analyzed its role in pathogenesis. The protein could be expressed by making use of its own signal peptide, so that Xyn11A accumulated in the culture medium, and could be purified by a simple protocol. The purified protein showed a curious heterogeneity in size, with at least seven isoforms differing slightly in size, typically with a difference of about 200 Da (Fig. 1). One may speculate that these differences in size may be accounted for by sugar monomers, and three *O*-glycosylation sites were indeed predicted for Xyn11A by the servers EnsembleGly http://turing.cs.iastate.edu/EnsembleGly and NetOGlyc 3.1 http://www.cbs.dtu.dk/services/NetOGlyc. Like other yeast and fungi, *P. pastoris *possesses an *O*-glycosylation system that can act on Ser and Thr residues of recombinant proteins, sometimes in residues not used by the original host and even if the original proteins are not normally glycosylated [25]. This system adds *O*-glycosaccharides composed of 1 to 4 mannose residues per glycosylation site [25], whose molecular weight is 180 Da, and therefore may be the cause of the differences in size for the purified enzyme. On the other hand, Xyn11A displays a putative propeptide [10] with a predicted molecular mass of 1494 Da, whose incomplete removal by *P. pastoris *could also introduce heterogeneity in the range observed in Fig. 1.

The molecular interaction between necrotrophic fungi, for which *B. cinerea *is becoming a model organism, and their host plants has suffered a paradigm shift in the last few years, that mainly makes the plant a much more active partner in the process than previously anticipated [1-3]. Instead of just being the passive target of fungal enzymes and toxic compounds causing the death of plant cells, evidences are being accumulated in favour of the participation of the PCD [26] in the plant-pathogen interaction, so that necrotrophic fungi's derived signals would induce the plant cells to kill themselves prior to their invasion. Here we show evidences that the xylanase Xyn11A produced by the necrotrophic plant pathogen *B. cinerea *is one of the signals that can induce necrosis in plants.

We showed previously that Xyn11A contributes significantly to virulence in this fungus, since the deletion of the corresponding gene from its genome causes a reduction in virulence [10], despite a modest reduction in xylanase activity for the mutant and the poor xylan content of the host plants. Now we have shown that 1) the xylan hydrolyzing activity of Xyn11A does not contribute to the infection process, since reintroducing in the *xyn11A *mutant altered variants of the *xyn11A *gene that code for enzymes lacking xylanase activity also restores the less-virulent phenotype back to the wild-type, as does the wild-type *xyn11A *gene; 2) the purified Xyn11A protein is able to induce necrosis in plants when infiltrated in leaves, and to induce one of the landmarks of HR, the production of ROS, and this ability is also independent of the catalytic activity since altered versions of the protein, unable to degrade xylan, also produce the same effects; and 3) exogenous application of Xyn11A to the infection process is also able to complement the *xyn11A *mutation, again even with Xyn11A variants with no xylan hydrolyzing activity. These three results imply that it is the necrosis-inducing activity, and not the ability to hydrolyze xylan, the main contribution of Xyn11A to virulence. This explains the apparent contradiction that this xylanase is required for full virulence in a pathogen that invades preferentially plant tissues which are poor in xylan, especially considering that *xyn11A *is one of five xylanase genes that can be found in the *B. cinerea *genome and that the *xyn11A *knock-out mutants show a modest 30% reduction in the xylanase activity secreted to the medium.

It has been clearly shown for the *Trichoderma viride *xylanase EIX that it causes a defence response in plants via recognition of the enzyme by a specific receptor (a leucine-rich repeat protein lacking the intracellular nucleotide binding domain) [20], internalization of the complex [27], induction of the second messengers nitric oxide and phosphatidic acid [28], and finally the form of programmed cell death known as the hypersensitive response which includes the production of ROS and the activation of defence genes [29]. It is clear, therefore, that xylanases are not causing plant cell death by a direct toxic effect, but by inducing the cells to kill themselves as a defence mechanism. Although an effective response defence against biotrophs, HR has been shown to facilitate *B. cinerea *infections [4] and it has been proposed that the fungus actively induces it. The similarity between the xylanases Xyn11A and EIX, both in their amino acid sequences (56% identity, 85% similarity) as well as in the appearance of the necrosis produced in tomato (Fig. 2C), seems to indicate that the mode of action of Xyn11A is identical to that explained above for EIX. If this is the case, the xylanase Xyn11A may be one of the means by which *B. cinerea *induces HR, so that this necrotroph would take advantage of the plants ability to recognize xylanase as a microbe associated molecular pattern, and generate a defence response aimed at biotrophic pathogens, to use the hypersensitive response for its own benefit. Xyn11A is, most probably, just one of the various means by which *B. cinerea *actively triggers the hypersensitive response in plants. Several small compounds have been identified that can cause necrosis when applied to plant tissues [6], which include the well studied compound botrydial and several less toxic compounds such as botcinolide. The symptoms caused in plants by the application of these isolated compounds resemble those caused by the fungus [30] and botrydial has been shown to be produced by the fungus *in planta *[31]. As far as we know, however, it is not known whether plant cells die as a result of a direct toxic effect of these compounds or by the induction of PCD. Another compound which has been implicated in PCD is oxalic acid. Although there are only indirect evidences [32] about the involvement of this compound in virulence for *B. cinerea*, it is known that this organism secretes high quantities of oxalic acid into the medium, both *in vitro *and *in planta *[1], and that the direct application of the compound to plant cells causes PCD [7]. Finally, *B. cinerea *secretes to the medium two isoforms of a toxic protein called Nep1 and Nep2, which also were reported to induce PCD in plants [9]. The xylanase Xyn11A seems to be, therefore, one of the various killing strategies that *B. cinerea *uses when invading its hosts, resulting in what has been called an "overkill" strategy [2] that allows this fungus to be such a successful pathogen with its enormous host range.

## Conclusions

The endo-β-1,4-xylanase Xyn11A contributes to the infection process in *B. cinerea *by inducing necrosis in the plant tissue. This necrosis-inducing activity of the enzyme is independent of the enzymatic, xylan-hydrolyzing activity and is located in a 30 amino acids peptide on the enzyme surface, which mediates binding to plant cells.

## Methods

### Biological material

All strains were routinely maintained at -80°C in 15% glycerol for long-term storage and at 4°C in silica gel [33] for routine use. *B. cinerea *wild-type strain B05.10 [34] was obtained from P. Tudzynski (Westfaelische Wilhelms-Universitaet Muenster, Germany). The new *xyn11A *knock-out mutant N23 was constructed as before, and characterized by PCR and Southern-blot as having a single integration of the transforming DNA in the *xyn11A *locus [10]. Conidia were prepared as described by Benito et al. (1998) from cultures on tomato-PDA plates (39 g of potato dextrose agar plus 250 g of homogenized tomato fruits per liter). Plant varieties used were tomato cv Moneymaker, tobacco cv. Havana, and two local tobacco varieties obtained from a local supplier, cv. Alcalá and cv. Paraíso.

### Expression of Xyn11A in *Pichia pastoris*

*xyn11A *ORF was amplified from *B. cinerea *cDNA with oligonucleotides XYL-F-BGL (5'-AGAAGATCTATGGTTTCTGCATCTTCC-3') and XYL-R-ECO (5'-AGAATTCCCCAGATTTAAGAAACAGTG-3'), digested with *Bgl *II+*EcoR *I and cloned at the same restriction sites of the *P. pastoris *plasmid pPIC3.5 (Invitrogen, http://www.invitrogen.com) behind the *AOX1 *promoter to generate plasmid pPICXYN. Xyn11A produced from this plasmid is translated from its own initiation codon and is secreted by making use of its own signal sequence. pPICXYN was linearized with *Sal *I and electroporated into *P. pastoris *using a Gene Pulser electroporator (Bio-Rad, http://www.bio-rad.com), following the manufacturers' instructions. The His^+ ^transformants were tested for the Mut^+ ^phenotype and grown in MX plates (0,34% yeast nitrogen base, 1% ammonium sulphate, 1% xylan, 0.5% methanol, and 4 × 10^-5^% biotin) to test for the secretion of xylanase by the production of a clear halo around the colonies, resulting from the degradation of xylan (Fig. 1A). Xyn11A secretion was also tested in liquid cultures for several transformants, and one of them, PICXYN18, was chosen to produce the enzyme by inducing for 48 hours in one of the media proposed by the manufacturer, BMMY, with the daily addition of 0.5% methanol. 200 ml of the supernatant from a culture in these conditions was used as starting material to purify Xyn11A. The enzyme was first precipitated with ammonium sulphate in the range of 45-80% saturation at 4°C, resuspended in 1 ml 50 mM sodium acetate pH 5.2, and loaded to a 40 × 1.6 cm sephacryl S-100 column equilibrated with 50 mM sodium acetate pH 5.2, 0.1 M NaCl. Three 2-ml fractions with xylanase activity were pooled and dialyzed overnight against 2 l of water at 4°C. When necessary, the purified protein was further concentrated by lyophilisation and resuspended in the appropriate volume of water. In the case of the mutant Xyn11A proteins devoid of xylanase activity, purifications were carried out in the same way except that SDS-PAGE, instead of activity assays, was used to test for the presence of the protein in the chromatographic fractions.

### Xylanase assay

Endo-β-1,4-xylanase activity was assayed by a modified version of the method of Bailey et al. [35]. Unless otherwise stated, reactions contained 1% Beechwood xylan in citrate-phosphate McIlvaine buffer [36], pH 5, plus the appropriate amount of enzyme in a final volume of 125 μl. Incubations were carried out at 35°C for 10 min and reactions were stopped by the addition of 187.5 μl of the dinitrosalicylic acid solution used to assay reducing sugars [35] and incubated 5 min in a boiling water bath. Finally, a DTX800 microplate reader (Beckman Coulter Inc., http://www.beckman.com) was used to read the absorbance of the samples at 540 nm. To determine optimal pH, assays were also made in McIlvaine buffer adjusted at pHs ranging from 3 to 7.

### Virulence and elicitation tests on leaves

To test the infectivity of *B. cinerea *strains, detached tomato or tobacco leaves were inoculated with 5-μl drops of 2 × 10^5 ^conidia per ml in TKKG solution (60 mM KH_2_PO_4_, 10 mM glycine, 0.01% Tween 20, 0.1 M glucose). The leaves were incubated at 22°C in a high-humidity chamber and the lesion areas were recorded daily. Necrosis inducing activity of purified proteins was assayed by infiltration into tomato or tobacco young leaves. Xylanase or elicitor epitope-GFP fusions, dissolved respectively in water or in 10 mM Tris pH 8, 30 mM NaCl, were forced into the leaves through stomata in the underside of the leaf with a 1-ml syringe without needle, so that the intercellular space became soaked in protein solution. The lesions were observed for up to one week after infiltration and the leaf remained attached to the plant during the whole experiment. Negative controls were regularly made by infiltrating water or buffer alone. Each infiltration assay was repeated at least three times. To detect H_2_O_2 _induction by xylanase in tomato leaves, one hour after infiltration the leaves were detached from the plant and the petioles were submerged in a solution of 1 mg/ml diaminobenzidine pH 3.8. After incubating for 16 hours at 22°C in an illuminated room, the leaves were boiled for 10 min in ethanol to eliminate chlorophyll and photographed.

### Site-directed mutagenesis of Xyn11A

The plasmid pMUTE was generated containing a 0,5-kb fragment carrying part of the *xyn11A *ORF and part of its terminator in a pBluescript KS+ backbone. This fragment was obtained by PCR with primers pNRXYNbFW (5'-TACACCGGATCCTACAAACC-3') and pNRXYNbRV (5'-GGAATTCGTGGCCAGGAACGAAATCG-3') and cloned in the *BamH*I and *EcoR*I restriction sites of plasmid pBluescript KS+. This plasmid was used as starting material for site-directed mutagenesis with the QuickChange Site Directed Mutagenesis kit (Stratagene, http://www.stratagene.com San Diego, California), using the following oligonucleotides: for the E122Q mutation, E122QFW (GGACTACCTCCCCCCTCATCCAGTACTACATCGTCG) and E122QRV (CGACGATGTAGTACTGGATGAGGGGGGAGGTAGTCC); for the E122S mutation, E122SFW (GGTTGGACTACCTCCCCCCTGATCAGCTACTACATCGTCG) and E122SRV (CGACGATGTAGTAGCTGATCAGGGGGGAGGTAGTCCAACC); for the E214Q mutation, E214QFW (CCAAATTGTTGCTGTTCAGGGTTACCAAAGCAGTGGATCCG) and E214QRV (CGGATCCACTGCTTTGGTAACCCTGAACAGCAACAATTTGG); and for the E214S mutation, E214SFW (CCAAATTGTGGCTGTTAGCGGTTACCAAAGCAGTGGTTCCGC) and E214SRV (GCGGAACCACTGCTTTGGTAACCGCTGACAGCCACAATTTGG). The insert of the resulting plasmids was completely sequenced to confirm the mutation and discard the presence of undesired mutations. The mutated *BamH*I-*EcoR*I *xyn11A *fragments were then transferred to the corresponding sites in pPICXYN, to generate the plasmids necessary to express the mutant Xyn11A proteins in *P. pastoris*. Additionally, *BamH*I-*Msc*I fragments from each mutated plasmid were exchanged for the corresponding fragment in pNRXYN, to transform the *B. cinerea xyn11A *knock-out mutant N23 and complement the mutation. pNRXYN was constructed by cloning the *Cla*I and *EcoR*I (blunted) fragment from plasmid pRXM [10] into the *Xba*I site (blunted) of pNR2 [37], and contains the whole *xyn11A *gene including promoter and terminator, as well as the nourseothricin resistance cassette.

### Expression of the 30-amino acids elicitor peptide in *Escherichia coli *and binding to tobacco protoplasts

A 90-bp region of *xyn11A *containing the putative elicitor epitope (residues 131 to 160 in the immature Xyn11A polypeptide) was amplified by PCR using oligonucleotides ELEPI-BGL (5'-GCAGATCTACCTACGATCCCTCCTCC-3') and ELEPI-KPN (5'-GCGGTACCGTTTGTACGGGTGGTCTCG-3'), which introduced the restriction sites *Bgl*II and *Xba*I, and cloned in the corresponding sites of pRSETB (Invitrogen, http://www.invitrogen.com) to generate the plasmid pEliX. This vector directs the expression of the peptide in *E. coli *fused to a poly-His tag to facilitate purification. The *mgfp4 *gene [38] was then amplified from the nos-GFP cassette [39] with either primer pairs GFP-BAM (5'-GCGGATCCGATGAGTAAAGGAGAAGAAC-3') and GFP-KPN (5'-GCGGTACCATGAGTAAAGGAGAAGAAC-3'), or GFP-BGL (5'-GCAGATCTGTATAGTTCATCCATGCC-3') and GFP-ECO (5'-GCGAATTCGCTTGACTCTAGCTTATTTG-3'), which introduced the restriction sites *BamH*I, *Kpn*I, *Bgl*II or *EcoR*I as indicated, for cloning into the corresponding sites of pEliX, so that two plasmids were generated, pGFPEliX and pEliXGFP, to direct the expression of the elicitor epitope in *E. coli *fused to either the carboxy or the amino terminus of GFP and to the poly-His tag. Tobacco protoplasts were prepared as [40], except that a concentration of 0.5% cellulase was used instead of 2%, and binding of the elicitor epitope-GFP fusions to them was assayed as [41]. Fluorescence microscopy was carried out with an Olympus BX-50 fluorescence microscope equipped with a U-MWIB filter to detect GFP and a U-MWIG filter to detect chlorophyll.

## Authors' contributions

NB generated and characterized the new *xyn11A *mutants, CG did the bioinformatics work, and JN did the rest of the experiments. NB and CG conceived the study and wrote the manuscript. All authors critically revised the manuscript, and all authors read and approved the final manuscript.
